# Emergence of Clade Ib Monkeypox Virus—Current State of Evidence

**DOI:** 10.3201/eid3108.241551

**Published:** 2025-08

**Authors:** Panayampalli S. Satheshkumar, Crystal M. Gigante, Placide Mbala-Kingebeni, Yoshinori Nakazawa, Mark Anderson, Stephen Balinandi, Sophia Mulei, James Fuller, Jennifer H. McQuiston, Andrea M. McCollum, Christina L. Hutson

**Affiliations:** Centers for Disease Control and Prevention, Atlanta, Georgia, USA (P.S. Satheshkumar, C.M. Gigante, Y. Nakazawa, M. Anderson, J. Fuller, J.H. McQuiston, A.M. McCollum, C.L. Hutson); Institut National de Recherche Biomédicale, Kinshasa, Democratic Republic of the Congo (P. Mbala-Kingebeni); Uganda Virus Research Institute, Entebbe, Uganda (S. Balinandi, S. Mulei)

**Keywords:** monkeypox virus, MPXV, clade Ia, clade 1b, clade II, mpox, outbreak, case-fatality rate, sexually transmitted infections, viruses, zoonoses

## Abstract

Mpox was first identified against the backdrop of the smallpox eradication campaign. Monkeypox virus (MPXV), the causative agent of mpox, has been maintained in animal reservoirs in the forested regions of West and Central Africa as 2 distinct clades; clade I has historically caused more severe infection in Central Africa than clade II, historically found in West Africa. However, rapid reemergence and spread of both MPXV clades through novel routes of transmission have challenged the known characteristics of mpox. We summarize mpox demographic distribution, clinical severity, and case-fatality rates attributed to genetically distinct MPXV subclades and focus on MPXV clade Ib, the more recently identified subclade. Broad worldwide assistance will be necessary to halt the spread of both MPXV clades within mpox endemic and nonendemic regions to prevent future outbreaks.

Monkeypox virus (MPXV), the causative agent of mpox (formerly known as monkeypox), has reemerged as a major public health concern across the globe because of recent outbreaks. Accordingly, the World Health Organization declared public health emergencies of international concern, initially for the global mpox outbreak on July 22, 2022, and again on August 14, 2024, for ongoing mpox outbreaks in the Democratic Republic of the Congo and nearby countries (*1*,*2*). MPXV has circulated in endemic regions since the 1970s, primarily because of zoonotic spillover followed by limited household transmission (*3*). However, more efficient spread in humans via sexual contact led to ≈100,000 mpox cases during May 2022–August 2024, including in 115 previously nonendemic countries (*4*). 

After eradication of smallpox (caused by variola virus) in 1980, MPXV has become the major orthopoxvirus infecting humans. The increased incidence of mpox has likely been caused by several factors, such as an increase in the proportion of immunologically naive populations after discontinuing routine smallpox vaccination and waning immunity in those persons previously vaccinated. Increased surveillance and diagnostic testing have also led to the detection of mpox in various countries after several decades with no reported cases (*5*,*6*).

MPXV is classified into 2 distinct clades and further into subclades on the basis of genetic differences (*7*). Historically, mpox cases caused by clade I and clade II MPXV were segregated to countries in central (clade I) and western (clade II) Africa, presumably because of geographic barriers separating the reservoir host(s) populations (Figure 1) (*8*). Clade I and II MPXV genomes differ by ≈0.4%–0.5% in nonrepetitive regions conserved between the clades and by the presence of 4 large insertion/deletions (*7*); the clades are estimated to have evolved separately over hundreds of years.

**Figure 1:**
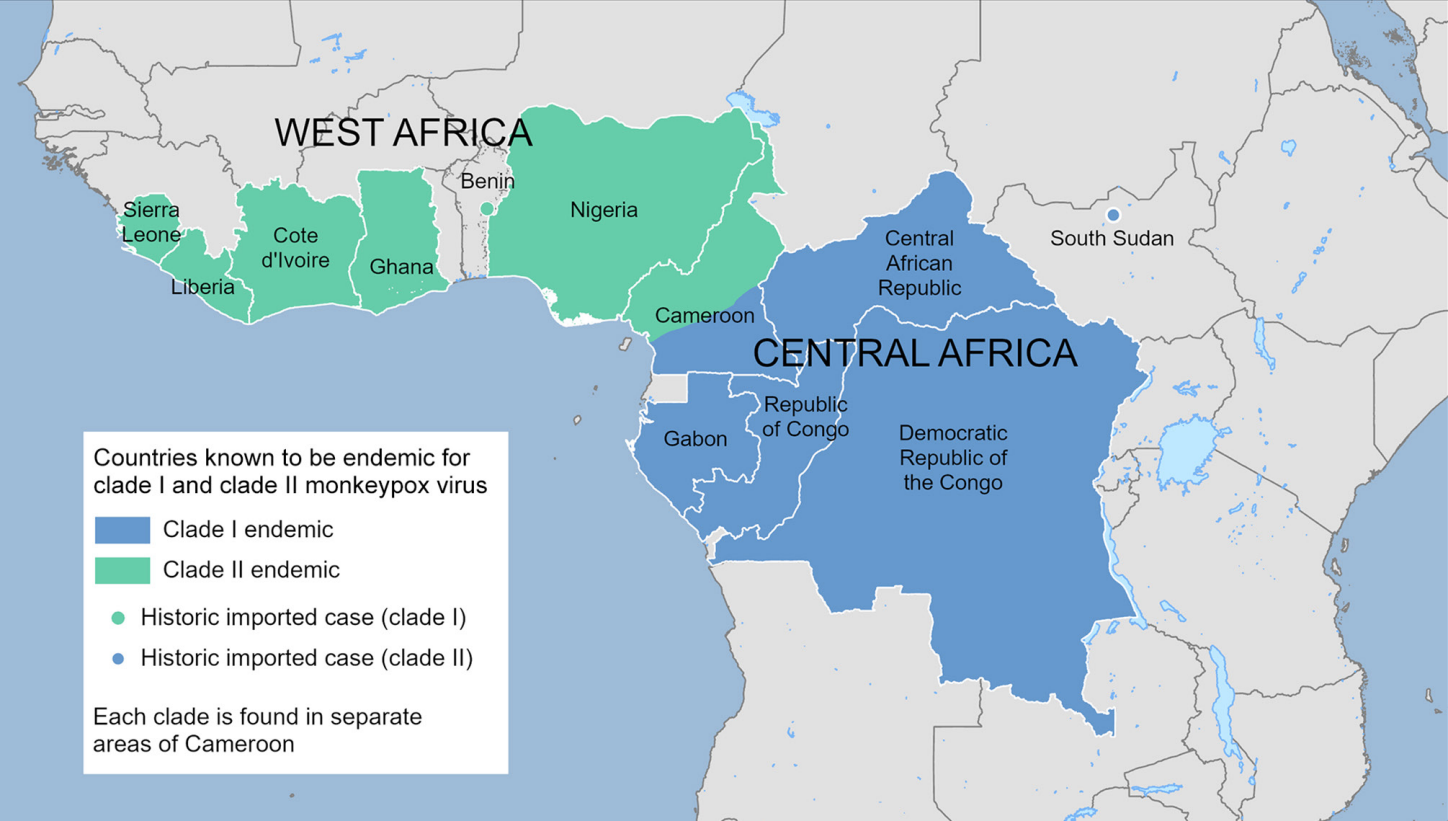
Geographic distribution of countries in Africa considered endemic for mpox caused by monkeypox virus clades I and II. Clade I (blue dot) and clade II (green dot) indicate historic imported mpox cases linked to mpox-endemic areas through known travel before 2022. Geographic separation of clades in Cameroon is approximate.

## Clade II MPXV

Clade II MPXV (previously known as West African MPXV) was first identified in the 1970s. According to sequence data, clade II MPXV is divided into subclades IIa and IIb. Before 2022, mpox cases caused by clades IIa and IIb were separated by the Dahomey Gap in West Africa (*9*), which might have been a geographic barrier for unknown MPXV reservoir hosts that enabled those 2 subclades to evolve separately (*8*,*9*). Clades IIa and IIb share ≈99.8% nucleotide identity in conserved nonrepetitive regions and can be differentiated by a large subclade-specific insertion/deletion. In 2017, Nigeria reported an mpox outbreak caused by MPXV clade IIb after decades of no reported cases (*10*). In 2022, clade IIb spread globally to many countries that had previously not reported mpox cases. Global mpox cases caused by clade IIb MPXV peaked for most countries during July–October 2022 (*11*), but the outbreak has been ongoing since then; low numbers of cases have been reported in many countries previously unaffected by mpox.

Since the 2017 outbreak in Nigeria, extensive human-to-human transmission of clade IIb MPXV has disproportionately impacted men who have sex with men through sexual activity (*10*,*12*,*13*), conflicting with the general understanding of mpox cases in endemic countries as being caused by zoonotic spillover followed by limited person-to-person spread. Moreover, starting with MPXV sequences from Nigeria in 2017 through the 2022 global outbreak, it became clear that clade IIb MPXV was accumulating mutations at a faster rate than previously reported for orthopoxviruses (*12*–*14*). Most single-nucleotide polymorphisms were GA to AA mutations, linked to the activity of host innate immunity proteins belonging to the apolipoprotein B mRNA editing catalytic polypeptide-like (APOBEC) 3 family (*12*–*14*). APOBEC3 family members exhibit diverse functions, including cytidine deamidation leading to signature G-to-A point mutations. The human genome encodes 6 functional APOBEC3 proteins, including APOBEC3F, which has been shown to produce extensive G-to-A mutations in the MPXV genome in cultured cells (*15*). APOBEC3 signature mutations are not observed in MPXV lineages having documented zoonotic transmission but are limited to those lineages involved in human-to-human transmission (i.e., clades Ia and IIa) (*12*,*13*); accumulations of APOBEC3-mediated signature mutations in MPXV lineages are now considered an indicator of sustained human-to-human transmission. It is not clear how those host-induced mutations are affecting MPXV; however, no evidence exists suggesting they cause increased virulence. The mutations have been reported across the genome in both coding and noncoding regions. In animal models, clade IIb viruses have been associated with decreased virulence compared with clades Ia and IIa (*16*), but genetic differences between those clades are not limited to APOBEC3-mediated mutations.

## Clade I MPXV

Whereas clade IIb MPXV spread globally in 2022 and 2023, mpox cases caused by clade I MPXV were reported only in known mpox endemic countries: the Democratic Republic of the Congo (DRC), the Republic of Congo, Gabon, Cameroon, and Central African Republic. None of the clade I–endemic countries reported cases caused by clade IIb MPXV during the 2022 global clade II outbreak except for Cameroon (only country in which clades I and II were both endemic before 2022), although virus characterization and testing were limited. Since 2022, the number of suspected clade I–associated mpox cases and deaths in DRC has increased considerably, and the highest number of suspected mpox cases in DRC was recorded in 2024 (*17*). Epidemiologic and genetic investigations of mpox in DRC during 2023–2024 revealed multiple outbreaks occurred involving different transmission dynamics, geographic distribution, and affected populations. 

Although mpox is endemic to DRC, in 2023 and 2024, mpox cases were being reported in previously disease-free areas in persons >15 years of age, whereas in other provinces those <15 years of age were most affected. Historically, children have been the most vulnerable to and affected by clade I–associated mpox in DRC; >80% of suspected cases and the highest case-fatality rates (CFRs) have been recorded in children <15 years of age (*3*). The historically high incidence of mpox in children was predominantly thought to be mediated by zoonotic spillover or occasional household transmissions (*18*). 

**Figure 2:**
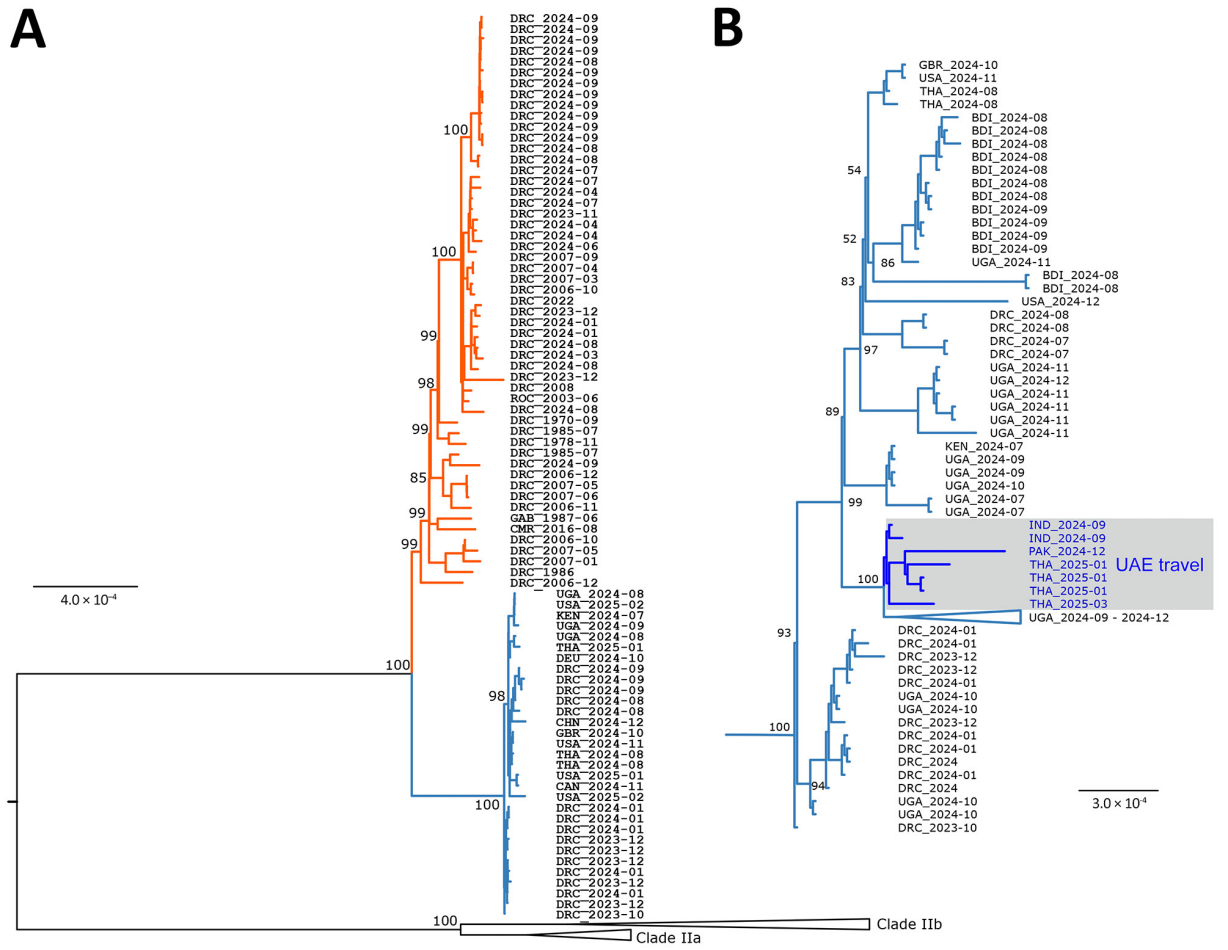
Phylogenetic analysis of clade I monkeypox virus (MPXV) sequences. A) Phylogenetic tree estimated for sequences from 1970–2024 according to core single-nucleotide differences among 103 clade I MPXV genomes. Sequences are identified by country (3-letter code) and collection date (year-month). Red branches indicate MPXV clade Ia; blue branches indicate clade Ib. Numbers at branch points indicate bootstrap support values (percentage of 1,000 replicates). B) Phylogenetic analysis of select clade Ib MPXV sequences. Blue text in gray box indicates sequences linked to persons who had traveled to UAE. Phylogenetic analyses were performed by using the maximum-likelihood method and the 3 substitution types model and equal base frequency plus empirical base frequencies plus proportion of invariable sites substitution model in ModelFinder (IQ-TREE version 2.2.6, https://iqtree.github.io). Whole-genome alignments of sequences downloaded from GISAID (https://www.gisaid.org) and GenBank (Appendix) databases were generated by using MAFFT version 7.490 (https://mafft.cbrc.jp/alignment/software). Trees were rooted by using clade II reference genomes: ON627808, ON585033, DQ011156, and DQ011153. Clade Ia sequences are not shown in the tree in panel B. Scale bars indicate nucleotide substitutions per site. BDI, Burundi; CAN, Canada; CHN, China; CMR, Cameroon; DEU, Germany; DRC, Democratic Republic of the Congo; GAB, Gabon; GBR, United Kingdom; IND, India; KEN, Kenya; PAK, Pakistan; ROC, Republic of Congo; THA, Thailand; UAE, United Arab Emirates; UGA, Uganda; USA, United States.

**Figure 3:**
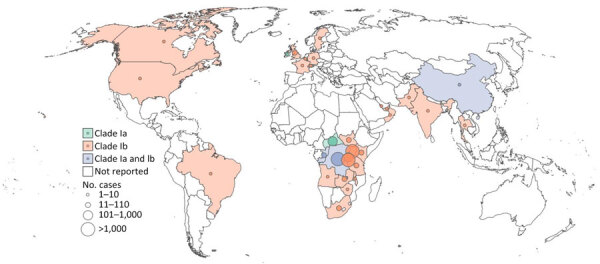
Global distribution of laboratory-confirmed cases of clade I monkeypox virus according to subclade during January 1, 2024–April 27, 2025 (*31*). Size of circles indicates estimated number of cases.

In late 2023, South Kivu, a province with few mpox cases, reported an mpox outbreak that had sexual contact as a primary factor for virus transmission (*19*). The MPXV causing cases in South Kivu was genetically distinct from clade I MPXV sequences from other regions of DRC (*19*,*20*), indicating that 2 distinct subclades were causing outbreaks in that country; the subclades have officially been designated Ia (which includes historical clade I MPXV sequences) and Ib (Figure 2, panels A, B) (*19*,*20*). Since late 2023, clade Ib MPXV has spread beyond South Kivu into other provinces of DRC and internationally to countries that had not previously reported mpox (Figure 3). The mpox outbreak caused by clade IIb continues to be associated with person-to-person transmission through both sexual and nonsexual close contact.

Although clade Ib MPXV has been recently discovered and named, similar MPXV sequences were collected during 2011–2012 in North and South Kivu (*21*), suggesting a clade Ib ancestor of the virus causing the 2023–2024 outbreak might have been present in the same region years earlier (*21*). Clade Ib MPXV sequences from the 2023–2024 outbreak are characterized by low overall genetic diversity, the accumulation of APOBEC3-mediated signature mutations, and a large 1,142-bp genomic deletion relative to clade Ia (*19*), resulting in loss of the complement control protein (CCP) gene. Although low genetic diversity within the clade Ib outbreak lineage is indicative of a recent outbreak, the abundance of APOBEC3-induced mutations is an indicator of sustained human-to-human transmission. However, transmission and demographic data for clade Ib suggests that increased sexual transmission, including through female sex trade workers, was predominantly driving the initial outbreak and subsequent sustained transmission (*22*). Clade Ia MPXV sequences from across DRC had much higher overall genetic diversity (≈10-fold higher than clade Ib) and a lower proportion of APOBEC3-induced signature mutations, suggesting that, unlike the clade Ib outbreak lineage, sustained human-to-human spread of clade Ia has not been pervasive throughout DRC, according to sequence data from 2018 through early 2024. Epidemiologic and genetic data for clade Ia MPXV still largely support the modality of zoonotic spillover followed by occasional human-to-human transmission or small outbreaks, but how the virus is repeatedly introduced into the human population is poorly understood.

One exception to the association between MPXV clade subtypes and zoonotic (clade Ia) or human-to-human (clade Ib) transmission has been reported in Kinshasa, DRC, where mpox outbreaks caused by both clade Ia and Ib MPXV are ongoing (*23*; T. Wawina-Bokalanga et al., unpub. data, https://doi.org/10.1101/2024.11.15.24317404). MPXV sequences from the Kinshasa outbreaks have accumulated APOBEC3-induced mutations (T. Wawina-Bokalanga et al., unpub. data), regardless of the clade subtype, reinforcing the notion that accumulation of APOBEC3-induced mutations is a molecular indicator of sustained human-to-human transmission. Since 2022, many investigations have focused on genetic changes that might have occurred in clade IIb MPXV that led to a global outbreak. Current evidence has not identified any subclade-specific virus adaptations that can explain why clade Ia, Ib, and IIb lineages have led to large outbreaks, suggesting that environmental factors (i.e., the network of persons into which the virus is introduced) play a critical role. Clade Ia, Ib, and IIb lineages have each caused outbreaks with sustained human-to-human transmission; thus, it is clear that any MPXV clade or subclade has the potential to cause such outbreaks, if (or when) the virus is introduced into a permissive transmission network that supports sustained transmission (*23*; T. Wawina-Bokalanga et al., unpub. data). Continued genomic sequence surveillance is critical to monitor ongoing mpox outbreaks in DRC and beyond.

## Comparisons of MPXV Clade Pathogenesis and Spread

Clinical studies of mpox in humans, as well as animal studies, have reported higher fatality rates for clade I than clade II infections; a higher number of persons with severe disease and more nonsexual human-to-human spread have also been reported for clade I MPXV (before the current outbreaks) compared with clade II infections (*7*,*16*,*24*,*25*). However, the pathogenesis, CFRs, and standards of treatment for mpox have varied for clades I and II. From a review of historic literature published before 2020, CFRs were estimated to be 10.6% for clade Ia and 3.6% for clade II MPXV infections without any medical intervention, and those estimates were likely influenced by limited or biased testing (*26*). Even in 2023, the CFR for clade I–associated suspected and confirmed mpox cases in DRC was estimated at 4.5% (*27*). However, general medical interventions have been shown to decrease CFR for MPXV clade I–associated cases. For instance, a 1.38% CFR was observed in the town of Kole during a natural history observational clinical study that included standard medical care (*28*), and a 1.7% CFR was observed with similar care during a clinical trial testing tecovirimat (both presumably clade Ia studies) (*29*). The mpox CFRs in South Kivu (which has only reported clade Ib MPXV infections) have consistently stayed <1% for suspected cases (*27*,*30*,*31*), lower than for clade Ia infections elsewhere in DRC. In addition, Burundi, which has had a clade Ib–associated mpox outbreak because of cross-border spread from DRC, had 1,607 confirmed mpox cases but only 1 fatality reported during July 22–October 31, 2024; moreover, in 1 Burundi study, ≈50% of mpox infections were in children, with no reported deaths (*31*–*33*). Taken together, those preliminary data suggest a lower CFR for clade Ib than for clade Ia, but additional studies are needed to confirm that difference. 

CFR estimates can be affected by differences in populations, demographics, and underlying health conditions (including food insecurity); access to healthcare; and testing biases. For example, the CFR (≈0.2% globally) for mpox caused by the clade IIb global outbreak lineage was considerably less than historical estimates for clade II, according to data from parts of the world with access to strong medical care systems. CFRs and clinical details from Kinshasa and other areas of DRC where clade Ia and Ib are co-circulating in comparable populations will provide a more reliable comparison of disease severity caused by those subclades (*4*).

Comparisons of MPXV genomic sequences revealed loss of the CCP gene (also known as monkeypox inhibitor of complement enzymes, encoded by *D14L*, a homologue of smallpox inhibitor of complement enzymes of vaccinia virus Copenhagen, encoded by *C4L*) in clades II and Ib compared with clade Ia. Researchers have hypothesized that deletion of the CCP gene might be the reason for decreased CFRs and disease severity historically reported for mpox caused by clades II and Ib compared with clade Ia viruses. However, animal studies investigating the effect of CCP gene loss have produced conflicting results. In 1 study, targeted deletion of the CCP gene from clade Ia MPXV (isolate ROC-2003-358) caused significantly decreased illness and death in infected prairie dogs, whereas addition of that gene to clade IIa MPXV caused slight changes in disease manifestation but had no apparent effect on disease-associated death (*34*). In another study, replacing large regions of the terminal genome of clade Ia with a corresponding sequence from clade IIa did not produce a difference in animal survival (*35*). A third study in nonhuman primates found differences in adaptive immune responses when the CCP gene was deleted from clade Ia MPXV (isolate Zaire-1979 005) (*36*). Investigators of all 3 studies concluded that the CCP deletion was not solely responsible for the differential pathogenicity between the clades (*34*–*36*). Additional animal studies with clade Ib isolates will help elucidate whether the genetic changes observed (CCP deletion and other mutations) do indeed result in a less virulent virus compared with clade Ia isolates. Other mutations (induced by APOBEC3 and non-APOBEC3 proteins) that distinguish clades Ia and Ib might also contribute to lower CFRs currently observed for clade Ib in South Kivu and Burundi compared with those of clade Ia cases elsewhere in DRC.

Even if clade Ib is confirmed to be less virulent than clade Ia, recent reports highlight the threat of that virus subclade, which appears to spread efficiently via sexual contact and within some household settings. Spread of clade Ib has occurred beyond South Kivu into neighboring and nonneighboring provinces of DRC, including the capital city Kinshasa, as well as internationally to multiple surrounding countries; >30 travel-associated cases have been reported outside of Africa. Because of privacy concerns, the country (or countries) to which patients had traveled was not released for most of those cases. However, only ≈17% had traveled to DRC; the remaining patients (≈80%) for which the US Centers for Disease Control and Prevention had received travel information had traveled to other countries in Africa that had sustained clade Ib outbreaks or to the United Arab Emirates (UAE). Eight mpox cases caused by clade Ib from 5 different countries (China, India, Oman, Pakistan, and Thailand) have been reported in persons who traveled to UAE. Sequences from 6 cases linked to UAE travel form a monophyletic cluster (Figure 2, panel B), suggesting a common ancestor. Considering the common travel history, the simplest explanation for clustering of those cases is exposure of the travelers to MPXV in UAE. Data suggest the presence of a similar strain of MPXV in Uganda and UAE around September/October 2024. Infections with the same strain in travelers to UAE during January–March 2025 warrant investigation into the possibility of sustained person-to-person transmission in UAE. Travelers should be aware of the exposure risks for clade Ib mpox in countries with ongoing clade I transmission (*37*).

## Mpox Diagnostics and Treatment

The genomic deletion characteristic of recent clade Ib sequences prevents detection by commonly used PCR developed for clade I (*38*). When performing clade-specific PCR that targets nonessential genes, laboratories should also use PCR targeting other genomic regions, including essential genes, such as the DNA polymerase gene (target of the Centers for Disease Control and Prevention nonvariola orthopoxvirus test) (*39*,*40*), to ensure mpox cases caused by clade Ib are not missed. In the United States, the recommended approach is to first (or concurrently) test by using a nonvariola orthopoxvirus or generic MPXV PCR targeting a conserved region. Then, after orthopoxvirus or MPXV is confirmed, additional clade-specific PCR or sequencing can be used (if needed) to determine the clade.

Irrespective of the differences in clades, the current smallpox and mpox vaccines are expected to be effective in controlling the spread and severity of disease because orthopoxviruses (e.g., MPXV [clades Ia, Ib, IIa, and IIb], vaccinia virus, variola virus) are >90% genetically related. Genetic relatedness was the premise and success behind smallpox eradication, which used vaccinia virus vaccine to cross-protect against smallpox. Live, replicating smallpox vaccines (e.g., ACAM2000; Emergent Bioservices, https://emergentbio.com) are contraindicated for immunocompromised persons and those with certain skin conditions. Hence, nonreplicating modified vaccinia Ankara vaccines, MVA-BN (Bavarian Nordic, https://www.bavarian-nordic.com) and JYNNEOS (https://jynneos.com), have been widely used in the United States and have been approved for use in adults >18 years of age. More than 1.2 million vaccine doses have been administered in the United States, and only very few vaccine breakthrough cases have been reported for most vaccinated persons after >2 years since completion of both doses (*41*). Those breakthrough case-patients also tended to exhibit milder disease course with no hospitalizations compared with unvaccinated patients (*42*). Multiple studies evaluating vaccine effectiveness for MVA-BN demonstrated >66%–86% protection against mpox (clade IIb); 2-dose vaccinations provided higher levels of protection than a single dose (*43*–*45*). To control clade I–associated mpox, several countries (DRC, Rwanda, Central African Republic, and Uganda) experiencing that outbreak have extended temporary or emergency use authorization of MVA-BN in adults, and vaccination of high-risk populations is ongoing. The European Medicines Agency approved MVA-BN for adolescents 12–17 years of age in late 2024, and the World Health Organization recently prequalified the vaccine for that same age group. In addition to MVA-BN, another smallpox vaccine, LC-16 (attenuated strain of vaccinia virus strain Lister; KM Biologics, https://www.kmbiologics.com), received emergency authorization in DRC. Unlike MVA-BN, LC-16 is a minimally replicating vaccine requiring 1 dose and approved for use in persons >1 year of age.

For many mpox patients in the United States, the smallpox antiviral drug tecovirimat (inhibits extracellular virus formation) has been administered as the primary therapeutic intervention through an expanded access investigational new drug protocol. For severe mpox cases or those patients at risk for severe disease (e.g., immunocompromised persons), combination therapy with tecovirimat and other antiviral drugs approved for smallpox treatment, such as cidofovir, brincidofovir (inhibits DNA replication), and vaccinia immune globulin, has been sometimes recommended on the basis of clinical needs of the patient (*46*). Prolonged treatment with tecovirimat has led to the development of resistant viruses; limited spread of resistant viruses among persons with no previous treatment has been observed, indicating the importance of containment and surveillance to detect those viruses (*47*,*48*). A randomized controlled study to determine the effectiveness of tecovirimat against clade I mpox in DRC reported no improvement in mpox resolution (*30*,*49*). A study on tecovirimat efficacy against clade IIb MPXV infections in the United States reported similar results (*50*). Additional studies assessing alternative drugs, earlier treatment with tecovirimat (i.e., before lesion onset), tecovirimat efficacy in immunocompromised persons, and combination treatment with other interventions are still needed. Because of the spread of mpox to many countries that historically did not see cases, including ongoing cases of clade IIb—associated mpox 2 years after the start of the outbreak, effective therapeutic agents for mpox are urgently needed.

## Conclusion

Mpox is an old disease but is now reemerging and causing international concern because of decreasing population immunity and sustained human-to-human transmission mediated through global travel, increased animal–human interfaces, and expansive sexual networks, leading to spread from small geographic regions and establishment of the disease in various parts of the world. Renewed global attention to mpox has occurred yet again because of the surge in reported mpox cases caused by clade I MPXV in DRC and the spread of the newly recognized clade Ib virus. Although clade Ib is in the spotlight, the remote forested regions of DRC where zoonotic clade Ia MPXV continues to circulate should not be forgotten. Broad worldwide assistance is necessary to halt the spread of both clade Ia and Ib within Africa to prevent future outbreaks.

Appendix GISAID and GenBank author acknowledgments for sequences used in phylogenetic analyses for emergence of clade Ib monkeypox virus—current state of evidence.

## References

[1] World Health Organization. WHO Director-General’s statement at the press conference following IHR Emergency Committee regarding the multi-country outbreak of monkeypox—23 July 2022. 2022 [cited 2025 May 9]. https://www.who.int/director-general/speeches/detail/who-director-general-s-statement-on-the-press-conference-following-IHR-emergency-committee-regarding-the-multi--country-outbreak-of-monkeypox--23-july-2022

[2] World Health Organization. WHO Director-General declares mpox outbreak a public health emergency of international concern. 2024 [cited 2025 May 9]. https://www.who.int/news/item/14-08-2024-who-director-general-declares-mpox-outbreak-a-public-health-emergency-of-international-concern PMC11376700 39218470

[3] Beer EM, Rao VB. A systematic review of the epidemiology of human monkeypox outbreaks and implications for outbreak strategy. PLoS Negl Trop Dis. 2019;13:e0007791. 10.1371/journal.pntd.0007791 31618206 PMC6816577

[4] Centers for Disease Control and Prevention. 2022–2023 Mpox outbreak global map [cited 2025 May 9]. https://archive.cdc.gov/#/details?url=https://www.cdc.gov/poxvirus/mpox/response/2022/world-map.html

[5] Reynolds MG, Wauquier N, Li Y, Satheshkumar PS, Kanneh LD, Monroe B, et al. Human monkeypox in Sierra Leone after 44-year absence of reported cases. Emerg Infect Dis. 2019;25:1023–5. 10.3201/eid2505.180832 30753125 PMC6478203

[6] Durski KN, McCollum AM, Nakazawa Y, Petersen BW, Reynolds MG, Briand S, et al. Emergence of Monkeypox - West and Central Africa, 1970-2017. MMWR Morb Mortal Wkly Rep. 2018;67:306–10. 10.15585/mmwr.mm6710a5 29543790 PMC5857192

[7] Likos AM, Sammons SA, Olson VA, Frace AM, Li Y, Olsen-Rasmussen M, et al. A tale of two clades: monkeypox viruses. J Gen Virol. 2005;86:2661–72. 10.1099/vir.0.81215-0 16186219

[8] Curaudeau M, Besombes C, Nakouné E, Fontanet A, Gessain A, Hassanin A. Identifying the most probable mammal reservoir hosts for monkeypox virus based on ecological niche comparisons. Viruses. 2023;15:727. 10.3390/v15030727 36992436 PMC10057484

[9] Nakazawa Y, Mauldin MR, Emerson GL, Reynolds MG, Lash RR, Gao J, et al. A phylogeographic investigation of African monkeypox. Viruses. 2015;7:2168–84. 10.3390/v7042168 25912718 PMC4411695

[10] Yinka-Ogunleye A, Aruna O, Dalhat M, Ogoina D, McCollum A, Disu Y, et al.; CDC Monkeypox Outbreak Team. Outbreak of human monkeypox in Nigeria in 2017-18: a clinical and epidemiological report. Lancet Infect Dis. 2019;19:872–9. 10.1016/S1473-3099(19)30294-4 31285143 PMC9628943

[11] World Health Organization. 2022–24 Mpox (monkeypox) outbreak: global mpox trends. 2024 [cited 2025 May 9]. https://worldhealthorg.shinyapps.io/mpx_global/#21_Epidemic_curves

[12] Gigante CM, Korber B, Seabolt MH, Wilkins K, Davidson W, Rao AK, et al. Multiple lineages of monkeypox virus detected in the United States, 2021-2022. Science. 2022;378:560–5. 10.1126/science.add4153 36264825 PMC10258808

[13] O’Toole Á, Neher RA, Ndodo N, Borges V, Gannon B, Gomes JP, et al. APOBEC3 deaminase editing in mpox virus as evidence for sustained human transmission since at least 2016. Science. 2023;382:595–600. 10.1126/science.adg8116 37917680 PMC10880385

[14] Isidro J, Borges V, Pinto M, Sobral D, Santos JD, Nunes A, et al. Phylogenomic characterization and signs of microevolution in the 2022 multi-country outbreak of monkeypox virus. Nat Med. 2022;28:1569–72. 10.1038/s41591-022-01907-y 35750157 PMC9388373

[15] Suspène R, Raymond KA, Boutin L, Guillier S, Lemoine F, Ferraris O, et al. APOBEC3F is a mutational driver of the human monkeypox virus identified in the 2022 outbreak. J Infect Dis. 2023;228:1421–9. 10.1093/infdis/jiad165 37224627 PMC11009509

[16] Americo JL, Earl PL, Moss B. Virulence differences of mpox (monkeypox) virus clades I, IIa, and IIb.1 in a small animal model. Proc Natl Acad Sci U S A. 2023;120:e2220415120. 10.1073/pnas.2220415120 36787354 PMC9974501

[17] World Health Organization. Regional Office for Africa. Mpox in the WHO African Region. Weekly regional situation report #17. 2024 Dec 15 [cited 2025 May 9]. https://iris.who.int/handle/10665/379903/AFRO-Mpox%20bulletin-%2015%20December%202024.pdf

[18] European Centre for Disease Prevention and Control. Outbreak of mpox caused by monkeypox virus clade I in the Democratic Republic of the Congo. 2024 [cited 2025 May 9]. https://www.ecdc.europa.eu/en/news-events/outbreak-mpox-caused-monkeypox-virus-clade-i-democratic-republic-congo

[19] Vakaniaki EH, Kacita C, Kinganda-Lusamaki E, O’Toole Á, Wawina-Bokalanga T, Mukadi-Bamuleka D, et al. Sustained human outbreak of a new MPXV clade I lineage in eastern Democratic Republic of the Congo. Nat Med. 2024;30:2791–5. 10.1038/s41591-024-03130-3 38871006 PMC11485229

[20] Masirika LM, Udahemuka JC, Schuele L, Ndishimye P, Otani S, Mbiribindi JB, et al. Ongoing mpox outbreak in Kamituga, South Kivu province, associated with monkeypox virus of a novel Clade I sub-lineage, Democratic Republic of the Congo, 2024. Euro Surveill. 2024;29:2400106. 10.2807/1560-7917.ES.2024.29.11.2400106 38487886 PMC10941309

[21] McCollum AM, Nakazawa Y, Ndongala GM, Pukuta E, Karhemere S, Lushima RS, et al. Human monkeypox in the Kivus, a conflict region of the Democratic Republic of the Congo. Am J Trop Med Hyg. 2015;93:718–21. 10.4269/ajtmh.15-0095 26283752 PMC4596588

[22] Masirika LM, Udahemuka JC, Schuele L, Nieuwenhuijse DF, Ndishimye P, Boter M, et al. Epidemiological and genomic evolution of the ongoing outbreak of clade Ib mpox virus in the eastern Democratic Republic of the Congo. Nat Med. 2025;31:1459–63. 10.1038/s41591-025-03582-1 39933565 PMC12092280

[23] Wawina-Bokalanga T, Akil-Bandali P, Kinganda-Lusamaki E, Lokilo E, Jansen D, Amuri-Aziza A, et al. Co-circulation of monkeypox virus subclades Ia and Ib in Kinshasa Province, Democratic Republic of the Congo, July to August 2024. Euro Surveill. 2024;29:2400592. 10.2807/1560-7917.ES.2024.29.38.2400592 39301745 PMC11484285

[24] Reed KD, Melski JW, Graham MB, Regnery RL, Sotir MJ, Wegner MV, et al. The detection of monkeypox in humans in the Western Hemisphere. N Engl J Med. 2004;350:342–50. 10.1056/NEJMoa032299 14736926

[25] Moss B. Understanding the biology of monkeypox virus to prevent future outbreaks. Nat Microbiol. 2024;9:1408–16. 10.1038/s41564-024-01690-1 38724757

[26] Bunge EM, Hoet B, Chen L, Lienert F, Weidenthaler H, Baer LR, et al. The changing epidemiology of human monkeypox-A potential threat? A systematic review. PLoS Negl Trop Dis. 2022;16:e0010141. 10.1371/journal.pntd.0010141 35148313 PMC8870502

[27] Government of the Democratic Republic of the Congo. Report on the epidemiological situation of monkeypox (Mpox) in the DRC—sitrep No 025 (29 August 2024) [cited 2025 May 9]. https://reliefweb.int/report/democratic-republic-congo/rapport-de-la-situation-epidemiologique-de-la-variole-simienne-mpox-en-rdc-sitrep-no-025-29-aout-2024

[28] Pittman PR, Martin JW, Kingebeni PM, Tamfum JM, Mwema G, Wan Q, et al.; Kole Human Mpox Infection Study Group. Clinical characterization and placental pathology of mpox infection in hospitalized patients in the Democratic Republic of the Congo. PLoS Negl Trop Dis. 2023;17:e0010384. 10.1371/journal.pntd.0010384 37079637 PMC10153724

[29] National Institutes of Health. The antiviral tecovirimat is safe but did not improve clade I mpox resolution in Democratic Republic of the Congo. 2024 [cited 2025 May 9]. https://www.nih.gov/news-events/news-releases/antiviral-tecovirimat-safe-did-not-improve-clade-i-mpox-resolution-democratic-republic-congo

[30] Brosius I, Vakaniaki EH, Mukari G, Munganga P, Tshomba JC, De Vos E, et al. Epidemiological and clinical features of mpox during the clade Ib outbreak in South Kivu, Democratic Republic of the Congo: a prospective cohort study. Lancet. 2025;405:547–59. 10.1016/S0140-6736(25)00047-9 39892407 PMC7618259

[31] World Health Organization. Global mpox trends. 2025 [cited 2025 May 9]. https://worldhealthorg.shinyapps.io/mpx_global/#severity

[32] Nizigiyimana A, Ndikumwenayo F, Houben S, Manirakiza M, van Lettow M, Liesenborghs L, et al. Epidemiological analysis of confirmed mpox cases, Burundi, 3 July to 9 September 2024. Euro Surveill. 2024;29:2400647. 10.2807/1560-7917.ES.2024.29.42.2400647 39421954 PMC11487916

[33] Nzoyikorera N, Nduwimana C, Schuele L, Nieuwenhuijse DF, Koopmans M, Otani S, et al. Monkeypox Clade Ib virus introduction into Burundi: first findings, July to mid-August 2024. Euro Surveill. 2024;29:2400666. 10.2807/1560-7917.ES.2024.29.42.2400666 39421956 PMC11487920

[34] Hudson PN, Self J, Weiss S, Braden Z, Xiao Y, Girgis NM, et al. Elucidating the role of the complement control protein in monkeypox pathogenicity. PLoS One. 2012;7:e35086. 10.1371/journal.pone.0035086 22496894 PMC3322148

[35] Earl PL, Americo JL, Reynolds S, Xiao W, Cotter C, Moss B. A functional approach to analyze the genetic basis for differences in virulence of monkeypox virus clades. Emerg Microbes Infect. 2025;14:2456144. 10.1080/22221751.2025.2456144 39824796 PMC11795743

[36] Estep RD, Messaoudi I, O’Connor MA, Li H, Sprague J, Barron A, et al. Deletion of the monkeypox virus inhibitor of complement enzymes locus impacts the adaptive immune response to monkeypox virus in a nonhuman primate model of infection. J Virol. 2011;85:9527–42. 10.1128/JVI.00199-11 21752919 PMC3165757

[37] Centers for Disease Control and Prevention. Prevention strategies for mpox, including vaccinating people at risk via sexual exposure, for U.S. travelers visiting countries with clade I mpox outbreaks. 2024 [cited 2025 May 9]. https://www.cdc.gov/han/2024/han00516.html

[38] Li Y, Zhao H, Wilkins K, Hughes C, Damon IK. Real-time PCR assays for the specific detection of monkeypox virus West African and Congo Basin strain DNA. J Virol Methods. 2010;169:223–7. 10.1016/j.jviromet.2010.07.012 20643162 PMC9628942

[39] Centers for Disease Control and Prevention. Non-variola orthopoxvirus generic real-time PCR test. 2022 [cited 2025 May 9]. https://www.cdc.gov/mpox/media/pdfs/2024/08/Non-variola-Orthopoxvirus-Generic-Real-Time-PCR-Test.pdf

[40] Li Y, Olson VA, Laue T, Laker MT, Damon IK. Detection of monkeypox virus with real-time PCR assays. J Clin Virol. 2006;36:194–203. 10.1016/j.jcv.2006.03.012 16731033 PMC9628957

[41] Centers for Disease Control and Prevention. Mpox vaccine administration in the U.S. 2024 [cited 2025 May 9]. https://archive.cdc.gov/#/details?url=https://www.cdc.gov/poxvirus/mpox/response/2022/vaccines_data.html

[42] Guagliardo SAJ, Kracalik I, Carter RJ, Braden C, Free R, Hamal M, et al. Monkeypox virus infections after 2 preexposure doses of JYNNEOS vaccine—United States, May 2022–May 2024. MMWR Morb Mortal Wkly Rep. 2024;73:460–6. 10.15585/mmwr.mm7320a3 38781111 PMC11115437

[43] Dalton AF, Diallo AO, Chard AN, Moulia DL, Deputy NP, Fothergill A, et al.; CDC Multijurisdictional Mpox Case Control Study Group. Estimated effectiveness of JYNNEOS vaccine in preventing mpox: a multijurisdictional case-control study—United States, August 19, 2022–March 31, 2023. MMWR Morb Mortal Wkly Rep. 2023;72:553–8. 10.15585/mmwr.mm7220a3 37200229 PMC10205167

[44] Deputy NP, Gerhart JL, Feldstein LR. Vaccine effectiveness against mpox in the United States. [Reply]. N Engl J Med. 2023;389:1440–1. 10.1056/NEJMc2309583 37819967 PMC10902861

[45] Wolff Sagy Y, Zucker R, Hammerman A, Markovits H, Arieh NG, Abu Ahmad W, et al. Real-world effectiveness of a single dose of mpox vaccine in males. Nat Med. 2023;29:748–52. 10.1038/s41591-023-02229-3 36720271 PMC9930701

[46] Rao AK, Schrodt CA, Minhaj FS, Waltenburg MA, Cash-Goldwasser S, Yu Y, et al. Interim clinical treatment considerations for severe manifestations of mpox—United States, February 2023. MMWR Morb Mortal Wkly Rep. 2023;72:232–43. 10.15585/mmwr.mm7209a4 36862595 PMC9997665

[47] Garrigues JM, Hemarajata P, Espinosa A, Hacker JK, Wynn NT, Smith TG, et al. Community spread of a human monkeypox virus variant with a tecovirimat resistance-associated mutation. Antimicrob Agents Chemother. 2023;67:e0097223. 10.1128/aac.00972-23 37823631 PMC10649028

[48] Smith TG, Gigante CM, Wynn NT, Matheny A, Davidson W, Yang Y, et al. Tecovirimat resistance in mpox patients, United States, 2022–2023. Emerg Infect Dis. 2023;29:2426–32. 10.3201/eid2912.231146 37856204 PMC10683829

[49] Ali R, Alonga J, Biampata JL, Kombozi Basika M, Maljkovic Berry I, Bisento N, et al.; PALM007 Writing Group. Tecovirimat for clade I MPXV infection in the Democratic Republic of Congo. N Engl J Med. 2025;392:1484–96. 10.1056/NEJMoa2412439 40239067 PMC12158442

[50] National Institutes of Health. NIH study finds tecovirimat was safe but did not improve mpox resolution or pain. 2024 [cited 2025 May 9]. https://www.nih.gov/news-events/news-releases/nih-study-finds-tecovirimat-was-safe-did-not-improve-mpox-resolution-or-pain

